# Disengagement from early psychosis intervention services: an observational study informed by a survey of patient and family perspectives

**DOI:** 10.1038/s41537-022-00300-5

**Published:** 2022-11-11

**Authors:** Alexia Polillo, Aristotle N. Voineskos, George Foussias, Sean A. Kidd, Sarah Bromley, Sophie Soklaridis, Wei Wang, Vicky Stergiopoulos, Nicole Kozloff

**Affiliations:** 1 Centre for Addiction and Mental Health, Toronto, ON Canada; 2 Department of Psychiatry, University of Toronto, Toronto, ON Canada

**Keywords:** Psychosis, Schizophrenia

## Abstract

Approximately one-third of patients with early psychosis disengage from services before the end of treatment. We sought to understand patient and family perspectives on early psychosis intervention (EPI) service engagement and use these findings to elucidate factors associated with early disengagement, defined as dropout from EPI in the first 9 months. Patients aged 16–29 referred to a large EPI program between July 2018-February 2020 and their family members were invited to complete a survey exploring facilitators and barriers to service engagement. A prospective chart review was conducted for 225 patients consecutively enrolled in the same EPI program, receiving the NAVIGATE model of coordinated specialty care, between July 2018-May 2019. We conducted a survival analysis, generating Kaplan–Meier curves depicting time to disengagement and Cox proportional hazards models to determine rate of disengagement controlling for demographic, clinical, and program factors. The survey was completed by 167 patients and 79 family members. The top endorsed engagement facilitator was related to the therapeutic relationship in both patients (36.5%) and families (43.0%). The top endorsed barrier to engagement was medication side effects in both patients (28.7%) and families (39.2%). In Cox proportional hazards models, medication nonadherence (HR = 2.37, 95% CI = 1.17–4.80) and use of individual psychotherapy (HR = .460, 95% CI = 0.220–0.962) were associated with early disengagement, but some of the health equity factors expected to affect engagement were not. Findings suggest that delivery of standardized treatment may buffer the effects of health disparities on service disengagement in early psychosis.

## Introduction

Early psychosis intervention (EPI) is a model developed to provide treatment early in the course of psychotic illness with the goal of improving patients’ long-term trajectories and reducing burden on young people and their families. While evidence suggests that young people with psychosis can achieve superior outcomes in EPI^1–4^, approximately one-third disengage from services prematurely^5,6^, with rates ranging from 12% to 53% due to variations in how disengagement was defined and length of follow-up^6,7^. Though there is no known minimum duration of EPI services required to achieve positive outcomes, evidence from randomized-controlled trials and real-world effectiveness studies in support of EPI have examined outcomes at 1–3 years. Few studies have examined early disengagement from services, presenting an opportunity to establish a consistent definition for it that considers timing, measures, quality, and extent of disengagement.

Considerable literature on EPI engagement is drawn from observational cohort studies producing inconsistent and contradictory results with respect to factors associated with disengagement from EPI services. To date, few factors, including a lack of family support, lower medication adherence, and problem substance use, have emerged as robust predictors of EPI disengagement^5,7^. However, most studies have used living with family as a proxy for family support, whereas patients may live with family members who are not involved in their mental health treatment or have substantial support from family members but do not reside together. Legal involvement has also been associated with disengagement, but not widely tested^5,7^. Other factors known to be associated with disengagement from general mental health services have not been adequately explored in early psychosis, including homelessness and race/ethnicity^7,8^, and may be of particular importance to programs in diverse urban centers.

Additional gaps relate to understanding disengagement from patient and family member perspectives^8^. Qualitative studies have elicited factors influencing engagement from patients, such as individualized care, program attributes, family influences, and personal characteristics^9,10^, with few studies exploring barriers to engagement. In a recent qualitative meta-synthesis, stigma, distressing experiences prior to care, inconsistencies between patient needs and treatment plan, patients’ desire to treat their condition without services, and duration and intensity of EPI care were cited as factors that may pose barriers to engagement later in treatment^9^. These themes warrant exploration in larger samples and could be used to explore factors that may contribute to disengagement early in care. Integrating chart review and survey methods to understand EPI disengagement may provide more insight into early disengagement and resolve inconsistencies from observational studies by eliciting feedback directly from patients and family members with lived experience and using this expertise to inform the observational study.

## Aims

We sought to understand patient and family perspectives on service engagement in an EPI program and use these findings to guide the investigation of factors associated with early disengagement in a cohort of patients in the same program. The current study had two objectives: (1) to understand patient- and family-reported facilitators and barriers to engagement and (2) to investigate factors associated with early disengagement from EPI services. Early disengagement was defined as dropout from services within the first 9 months of treatment. We hypothesized that a lack of family support, lower medication adherence, and problem substance use would be associated with an increased risk of early disengagement from EPI services^5,7,11^. We also explored a relationship between homelessness, legal involvement, and race/ethnicity with early disengagement.

## Method

### Overview and study design

The Centre for Addiction and Mental Health (CAMH) in Toronto, Canada houses the largest EPI program in the country. Based on Ontario EPI Program Standards, it aims to provide comprehensive treatment delivered by a multidisciplinary team early in the course of illness^12^. The program provides consultation for young people up to age 29 with provisional psychosis and offers up to three years of treatment for individuals aged 16–29 with affective, non-affective, and substance-induced psychosis. Patients who are appropriate for EPI but have a closer service are bridged to their local program. CAMH uses the NAVIGATE model of coordinated specialty care, consisting of four core treatment services: individual resiliency training (IRT), supported employment and education (SEE), family education, and individualized medication management^12,13^.

Patients are considered to have disengaged from the program if they are formally discharged or did not attend appointments for a period of three months or more, despite still requiring ongoing treatment as assessed by their clinician. Efforts to engage a patient prior to considering them disengaged are individualized to the patient but generally include multiple methods of contact (phone, email, or through family members with consent) over a period of 3 months. Patients are finally sent a letter informing them that they will be discharged if there is no further contact within the next month.

For the survey, we invited people aged 16–29 referred to the program between July 2018-February 2020 and their family members to complete a survey exploring facilitators and barriers to engagement in EPI services. A prospective chart review was conducted of the records of 225 patients who were consecutively enrolled in EPI services at CAMH and attended at least one follow-up appointment between July 1, 2018-May 6, 2019. This observational study is reported according to the STROBE guidelines (Supplementary Table S1)^14^. The sample size was determined to have sufficient power (80%) to detect risk factors with a hazard ratio as low as 1.91 with an overall disengagement rate of 33.3% and a hazard ratio of 2.26 with a disengagement rate of 20%. All procedures involving human subjects/patients were approved by the Research Ethics Board (REB) at CAMH. Participants provided informed consent electronically for surveys, but chart review consent was not required as per CAMH’s REB.

### Survey

#### Survey Procedures

We used Research Electronic Data Capture (REDCap), a secure web-based platform with built-in tools for data collection and storage, to send web-based consent forms and surveys to patients and families referred to EPI services^15–17^. Participants who provided verbal consent to the clinic administrator were sent an electronic consent form by email or text message, or approached and consented in person. Web-based surveys were sent automatically 30 days after participants consented to the study to capture early experiences in the program, with follow-up email and phone reminders. Participants were compensated with a $10 gift card or e-gift card.

#### Survey questions

Participants were asked about demographic factors based on a standardized health equity tool^18^, service utilization, facilitators and barriers to engagement in mental health treatment, and suggestions for improving EPI service engagement. Items relevant to EPI engagement were chosen from validated tools that measure aspects of service engagement, including the Service Engagement Scale (items were adapted to be patient-rated rather than clinician-rated)^19^, the Working Alliance Inventory (WAI)^20^, and Scale To Assess the Therapeutic Relationship in Community Mental Health Care: Patient Version (STAR-P)^21^, as well as other aspects of “youth-friendly” services identified in the literature^22^. Respondents were asked to check all facilitators and barriers that applied to them and then rank their top 5.

### Observational study

#### Observational study procedures

Trained research staff extracted data from CAMH’s electronic health record into a structured REDCap database at three months (representing the first three months of treatment) and 9 months. Every twentieth chart was extracted by the principal investigator and two research staff to assess interrater reliability, which ranged from moderate to almost perfect (Gwet’s AC, 0.43–1.00 and intraclass correlation, 0.98)^23,24^.

#### Observational study variables

The primary outcome of early disengagement, defined as dropout from services within the first nine months of EPI treatment, was distinguished from all-cause discharge, which included transitions to local EPI programs or other mental health services. Our definition of early disengagement was clinician-defined and based on the structure of CAMH’s EPI program and delivery of the NAVIGATE model. Services are offered to patients for up to three years with the majority of NAVIGATE content delivered in the first two years of treatment, and core modules offered during the first year. Patients in the study were coded as having disengaged from services if they were formally discharged or did not attend appointments for a period of three months or more, despite still requiring ongoing treatment as assessed by their clinician^11^. For patients who disengaged for other reasons, data were censored at the time of patient death, move to another country, or transfer to EPI or other local mental health services. Data were also censored for patients still engaged in the program at nine months.

Most baseline demographic variables were extracted from CAMH’s standardized Health Equity Form routinely completed by patients at their first appointment^18^. Demographic variables included age, gender, self-reported racial/ethnic group, country of birth, living situation, employment status, and highest level of education. Additional variables were extracted from clinician documentation, including any documented experience of homelessness, legal involvement, family involvement in care, problem substance use, diagnoses, referral source, and distance to the clinic calculated as the number of kilometres between participant’s home address and the clinic using postal codes. Use of IRT and SEE, as well as medication nonadherence, defined by any documentation of nonadherence among patients who were taking antipsychotic or mood-stabilizing medication, were captured through clinical documentation in the first 3 months of care. Recoded variables are outlined in Supplementary Table S2.

### Statistical Analysis

Descriptive statistics were calculated for demographic characteristics of patients and family members who completed the survey, and proportion of patients and family members who endorsed each facilitator, barrier, and suggestion to improve engagement. Descriptive statistics were also calculated for the full sample of patients in the observational study and based on engagement status.

We modelled time-to-disengagement, calculated as the number of days from first treatment encounter to the day patients were last actively engaged in treatment, using a Kaplan–Meier curve. In addition to age and gender, we selected variables thought to be associated with disengagement based on past EPI literature (family involvement in care, problem substance use)^5,7^ and general mental health literature (racial/ethnic group, homelessness) a priori. Employment status, legal involvement, living alone, and medication nonadherence were added to the model based on emergent evidence of their association with EPI disengagement^7^. Based on survey findings highlighting the importance of the therapeutic relationship, we examined use of two components of NAVIGATE in the first 3 months of care, IRT and SEE. IRT is delivered by a clinician who also provides case management, which is sometimes prioritized early in care. SEE is typically offered as early as possible in treatment. Survey findings lent support to test the relationship between medication nonadherence and disengagement, as well as distance to services.

Although our study integrated two types of quantitative methods, we used a traditional mixed methods analysis approach: an exploratory sequential design to test effects of variables identified in the survey as key facilitators and barriers to engagement and a cross-case matrix to identify complementary and contradictory findings (reported in Table 1)^25,26^.

**Table 1:** Cross-case matrix depicting how survey results informed chart review variable selection.

Facilitators and barriers from surveys	Themes	Chart review variables
• My clinician speaks with me about my personal goals and thoughts about treatment	Therapeutic relationship	Early use of IRT and SEE
• My clinician and I agree on what is important for me to work on		
• I believe my clinician has an understanding of what my experiences have meant to me		
• My family member has a positive impression of the clinician(s)		
• My family member believes that the treatments are helpful or will be helpful • My family member does not like/trust the clinician		
• I help my family member with reminders, transportation, etc.	Family involvement	Living without family Family involvement in care
• My own motivation and commitment to treatment • My family member is motivated and committed to treatment • I feel uncomfortable leaving my house or going to unfamiliar places • I forget appointments or lose track of time	Individual factors	(No related variable measured)
• My family member is bothered by medication side effects • I am bothered by medication side effects	Medication side effects	Medication nonadherence
• I don’t like coming to a hospital • Past negative experiences with services	Stigma	(No related variable measured)
• Location of Services • Times services are offered	Practical challenges	Distance from services

Log-rank tests and Cox proportional hazards models were used to examine the relationship between selected variables and rate of early disengagement after checking the proportionality of the hazard function over time assumption using statistical tests and graphical diagnostics of Schoenfeld residuals. The most prevalent category was used as the reference group. Univariable tests were performed with covariates independently to obtain the univariable hazard ratios. Covariates that were statistically significant at an a priori level of *p* < 0.25 were retained in the final multivariable analysis. Analyses were conducted using Stata statistical software^27^. Responses were excluded for patients who indicated declined, don’t know, or prefer not to answer on structured health equity questions. All statistical tests were two-tailed and considered statistically significant at a *p* value < 0.05.

## Results

### Survey

A total of 447 patients and 187 family members agreed to receive the consent form, with 48.3% (*n* = 216) of patients and 58.3% (*n* = 109) of family members consenting to participate in the study. Of those who consented, 77.3% (*n* = 167) of patients and 72.5% (*n* = 79) of family members completed the survey^28^. Patients were a mean age of 22.8 ± 3.5 years, 45.8% were male, 64.1% lived with family, 37.0% were white, 58.3% were vocationally active, and 63.5% were born in Canada. Family members were a mean age of 47.8 ± 12.6, 59.5% were mothers, 72.2% lived with the identified patient, and 70.9% completed postsecondary education (Table 2).

**Table 2:** Survey demographic characteristics for patients and family members.

Variable	Patients (*n* = 167)	Family (*n* = 79)
	*n* (%)	
Age in years, (M ± SD)	22.8 ± 3.46	47.8 ± 12.57
Gender^a^		
Male	76 (45.8)	18 (22.8)
Female	78 (47.0)	61 (77.2)
Trans, non-binary, two-spirit^b^	12 (7.2)	0 (0)
Relationship to patient (mother)	–	47 (59.5)
Racial/ethnic group^c,d^		
Asian	41 (24.9)	12 (15.4)
Black	23 (13.9)	13 (16.7)
White	61 (37.0)	41 (52.6)
Other visible minority^e^	40 (24.2)	12 (15.4)
Born in Canada	106 (63.5)	40 (50.6)
Living with family	107 (64.1)	–
Living with patient	–	57 (72.2)
In a relationship	29 (17.4)	49 (62.0)
Vocationally active^f,g^	95 (58.3)	66 (84.6)
Highest level of education^h^		
High school or less	56 (33.9)	8 (10.1)
Attended postsecondary	70 (42.4)	15 (19.0)
Completed postsecondary	39 (23.6)	56 (70.9)

*n* sample size, *M* mean, *SD* standard deviation.

^a^1 response was “don’t know.”

^b^Categories combined due to small cells.

^c^1 response was “don’t know” and 1 patient declined to answer.

^d^1 family member declined to answer.

^e^Other visible minority include Middle Eastern, Indian-Caribbean, First Nations, Metis, Latin American, mixed heritage, and other racial/ethnic groups not included in the list.

^f^3 patients declined to answer and 1 responded “don’t know.”

^g^1 family member declined to answer.

^h^1 patient declined to answer and 1 responded “don’t know.”

#### Top facilitators

Top patient-reported facilitators of engagement related to the therapeutic relationship, with patients highlighting the importance of feeling understood by their clinician (36.5%, *n* = 61; Supplementary Table S3) and agreeing on a treatment plan (34.1%, *n* = 57), as well as having discussions about personal goals and thoughts about treatment (43.7%, *n* = 73). Other top facilitators included patients’ self-reported motivation and commitment to treatment (41.9%, *n* = 70) and location of EPI services (35.3%, *n* = 59). Similarly, top family-reported facilitators were patients having a positive impression of the clinician (43.0%, *n* = 34), their level of motivation and commitment to treatment (36.7%, *n* = 29), believing treatments are helpful (36.7%, *n* = 29), location of EPI services (32.9%, *n* = 26), and having help from family members with transportation and appointments reminders (34.2%, *n* = 27).

#### Top barriers

Medication side effects was the top patient- and family-reported barrier to engagement, endorsed by 28.7% (*n* = 48) and 39.2% (*n* = 31) respectively. Forgetting or losing track of appointments (25.7%, *n* = 43), stigma related to coming to a hospital (24.0%, *n* = 40), past experiences with services (21.0%, *n* = 35), feeling uncomfortable leaving the house or going to an unfamiliar place (18.6%, *n* = 31), and location of services (18.6%, *n* = 31) were also identified as barriers to engagement by patients. Other top family-reported barriers included location of services (26.6%, *n* = 21), times services are offered (19.0%, *n* = 15), patients wanting to address problems without professional help (16.5%, *n* = 13), and disliking or not trusting the clinician (17.7%, *n* = 14).

#### Suggestions for improvements

Both patients and family members suggested their engagement in services could be improved with evening (*n* = 54, 32.3% and *n* = 30, 38.0%) and weekend appointments (*n* = 57, 34.1% and *n* = 36, 45.6%), as well as appointment reminders (*n* = 61, 36.5% and *n* = 43.0%; Supplementary Table S4). Patients cited text message (*n* = 46, 27.5%) and email (*n* = 49, 29.3%) communication to improve engagement, while family members suggested text message (*n* = 29, 36.7%) communication and more involvement of family members and other supports in treatment (*n* = 34, 43.0%).

### Observational cohort

**Fig. 1:**
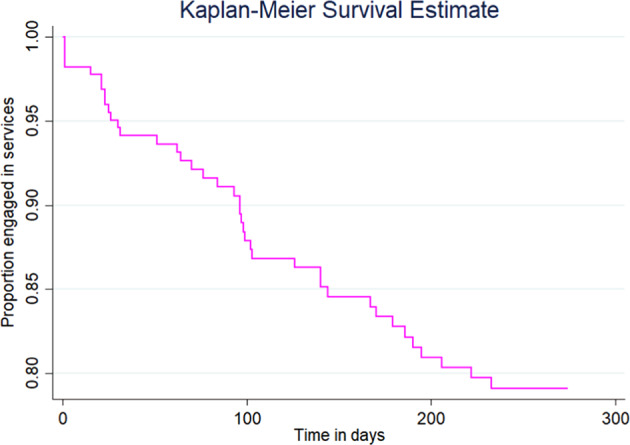
Kaplan–Meier curve modelling the proportion of total patients (*n* = 225) who are still engaged in services by time in days. Data were censored at the time of discharge for reasons other than disengagement, including death, move to another country, or transfer to EPI or other local mental health services. Data were also right-censored for patients still engaged in the program at 9 months.

Baseline characteristics are described in Table 3. Of the full sample of 225 patients, 44.4% (*n* = 100) of patients were not in treatment at 9 months. Approximately one quarter (26.7%; *n* = 60) of patients were transferred to EPI or other mental health services closer to home, had died, or had moved out of the country, and 17.8% (*n* = 40) dropped out of EPI services early. Median time to dropout for those who disengaged early was 94.5 days (IQR = 25.5–142) or approximately 3 months (Fig. 1).

**Table 3:** Baseline demographics, clinical characteristics and service use history of patients in the observational cohort by engagement status.

Variable	Full sample (*n* = 225)	Engaged or other (*n* = 185)	Early disengaged (*n* = 40)
	*n* (%)		
Age in years, (M ± SD) Age in years, (median (IQR))	22.6 ± 3.21 23 (20–25)	22.7 ± 3.25 23 (20–25)	22.3 ± 3.01 22 (20–24)
Gender (female or other)^a,b^	69 (30.8)	56 (30.4)	13 (32.5)
Racial/ethnic group^c^			
Asian	61 (28.2)	55 (31.1)	6 (15.4)
Black	44 (20.4)	36 (20.3)	8 (20.5)
White	62 (28.7)	49 (27.7)	13 (33.3)
Other visible minority^d^	49 (22.7)	37 (20.9)	12 (30.8)
Born in Canada^e^	135 (61.6)	113 (62.8)	22 (56.4)
Living without family	60 (26.7)	50 (27.0)	10 (25.0)
Experienced homelessness	47 (20.9)	35 (18.9)	12 (30.0)
Vocational activity			
Full/part time work	50 (22.2)	40 (21.6)	10 (25.0)
Full/part time school	48 (21.3)	39 (21.1)	9 (22.5)
Unemployed	127 (56.4)	106 (57.3)	21 (52.5)
Highest level of education^f^			
High school or less	77 (35.0)	60 (33.3)	17 (42.5)
Attended some postsecondary	104 (47.3)	83 (46.1)	21 (52.5)
Completed postsecondary	39 (17.7)	37 (20.6)	2 (5.0)
Legal involvement	44 (19.6)	31 (16.8)	13 (32.5)
Family involvement in care	154 (68.4)	128 (69.2)	26 (65.0)
Problem substance use	107 (47.6)	87 (47.0)	20 (50.0)
Non-affective psychosis	160 (71.1)	129 (69.7)	31 (77.5)
Acute referral source (inpatient unit or emergency department)	145 (64.4)	121 (65.4)	24 (60.0)
Medication nonadherence in first 3 months			
No	113 (50.2)	99 (53.5)	14 (35.0)
Yes	105 (46.7)	81 (43.8)	24 (60.0)
Never started medication	7 (3.1)	5 (2.7)	2 (5.0)
Use of IRT in first 3 months	113 (50.2)	102 (55.1)	11 (27.5)
Use of SEE in first 3 months	89 (39.6)	80 (43.2)	9 (22.5)
Distance to services in km, (M ± SD)^g^ Distance to services in km, (median (IQR))	16.4 ± 28.4 12 (5–21)	16.3 ± 30.8 11 (5–19)	17.0 ± 11.3 15 (7–23)

*n* sample size, *M* mean, *SD* standard deviation, *IQR* interquartile range, *EPI* early psychosis intervention, *IRT* individual resiliency training, *SEE* supported employment and education, *km* kilometers.

^a^Other gender categories combined with female due to small cells.

^b^response was “do not know.”

^c^responses were “prefer not to answer” and 5 were “don’t know.”

^d^Other visible minority groups include Middle Eastern, Indian-Caribbean, First Nations, Indigenous/Aboriginal, Latin American, and mixed heritage.

^e^responses were “don’t know.”

^f^Highest level of education could not be determined for five individuals.

^g^Postal codes could not be determined for three individuals.

Results of the log-rank tests are reported in Supplementary Table S5 and the Cox proportional hazards models are reported in Table 4. In univariable models, having legal involvement (HR = 2.24, 95% CI = 1.16–4.35) was associated with an increased risk of early disengagement, while early use of IRT (HR = 0.310, 95% CI = 0.155–0.621) and early use of SEE (HR = 0.366, 95% CI = 0.174–0.770) were associated with a decreased risk of disengagement in the first nine months of treatment. Having a documented experience of homelessness (HR = 1.55, 95% CI = .789–3.05), identifying as Asian (HR = 0.430, 95% CI = 0.163–1.13), and early medication nonadherence (HR = 1.90, 95% CI = 0.984–3.68) met the threshold for inclusion in our multivariable model. In the multivariable model, only early medication nonadherence (HR = 2.37, 95% CI = 1.17–4.80) and early use of IRT (HR = 0.460, 95% CI = 0.220–0.962) had a statistically significant association with disengagement, while the effect of early use of SEE (HR = 0.457, 95% CI = 0.200–1.04) approached statistical significance.

**Table 4:** Cox proportional hazards models for disengagement from early psychosis intervention.

	Univariable			Multivariable		
Variable	HR	95% CI	*P*	HR	95% CI	*P*
Gender (female or other)	1.06	0.549–2.06	0.855	–	–	–
Age	.951	0.862–1.05	0.314	–	–	–
Racial/ethnic group:						
Asian	0.430	0.163–1.13	**0.087**	0.433	0.161–1.16	0.096
Black	0.756	0.313–1.82	0.534	0.699	0.274–1.78	0.453
White (ref)	–	–	–	–	–	–
Other visible minority	1.14	0.522–2.51	0.737	1.11	0.475–2.58	0.812
Experienced homelessness	1.55	0.789–3.05	**0.203**	0.792	0.340–1.85	0.590
NEET	0.830	0.446–1.54	0.555	–	–	–
Living without family	0.900	0.440–1.84	0.773	–	–	–
Legal involvement	2.24	1.16–4.35	**0.017**	2.02	0.889–4.59	0.093
Family involvement in care	0.841	0.439–1.61	0.603	–	–	–
Problem substance use	1.20	.647–2.24	0.558	–	–	–
Early medication nonadherence	1.90	0.984–3.68	**0.056**	2.37	1.17–4.80	**0.017**
Early use of IRT	0.310	0.155–0.621	**0.001**	0.460	0.220–0.962	**0.039**
Early use of SEE	0.366	0.174–0.770	**0.008**	0.457	0.200–1.04	0.063
Distance from program (in km)	1.00	0.992–1.01	0.869	–	–	–

*HR* hazard ratio, *CI* confidence interval, *ref* reference category, *NEET* not engaged in employment, education, or training, *IRT* individual resiliency training, *SEE* supported employment and education, *km* kilometers.

*P* in bold indicates statistical significance.

## Discussion

In a large, urban EPI program delivering a standardized model of coordinated specialty care, we found that almost one-fifth of patients disengaged early. Two factors traditionally associated with disengagement among young people with psychosis—problem substance use and lack of family support—were not associated with early disengagement. Additional equity-related factors, including homelessness and race/ethnicity, were similarly not found to be associated with early disengagement. Instead, adherence to specific components of the NAVIGATE model in the first 3 months of care, particularly medication and IRT, predicted disengagement at 9 months of treatment. Similar themes were echoed in the surveys, with patients and families endorsing medication side effects as a top barrier and the therapeutic relationship as a top facilitator to engagement. Appointments outside of business hours, use of reminders, and communication leveraging technology, including text message and email, were endorsed by patients and family members as suggestions to improve engagement in EPI services. These findings point to specific factors that can be identified and addressed early in care to improve engagement and suggest that structured models of care may buffer effects of traditional factors found to be associated with disengagement.

Our early disengagement rate is in the lower range of those found in a recent systematic review, which identified rates ranging between 12% and 53%^7^. However, inconsistencies in the way disengagement is assessed and length of study follow-up can make it difficult to draw accurate comparisons across studies and contributes to inconclusive findings about which factors best predict disengagement^7,29^. Qualitative research can help unpack the complexity of EPI disengagement. A qualitative study identified changing priorities, perspectives on treatment, needs, and levels of autonomy as factors that help shape a patient’s decision to disengage from EPI services^30^. These factors move beyond traditional indicators of engagement and can help us better define and understand it; however, more qualitative research is needed.

The definition of disengagement in past studies also does not differentiate between patients who dropped out of services and those who were discharged for other reasons, including transitioning to services closer to home^7^, potentially inflating the number of patients who disengaged. Past studies have also focused on longer term disengagement in the first 2 to 3 years of treatment, with little attention given to early disengagement. Our focus on patients who have dropped out of EPI services in the first 9 months may have better potential to improve service retention early in care.

In contrast to previous studies, we did not find that family support, defined by living with family or family involvement in care, was associated with early disengagement from EPI services. NAVIGATE clinicians proactively engage family members early in treatment^13^, which may explain the high rates of family involvement in our study. It is possible that using a low threshold for determining family support diluted the impact that might be observed with higher-quality family engagement. Similarly, no association was found between problem substance use and early disengagement. Substance use education and coping skills provided through IRT modules may have mediated the risk of disengaging for those with problem substance use through harm reduction principles^31–33^. Legal involvement was found to predict disengagement in univariable models, but was better explained by clinical and service use factors in multivariable models. Patients navigating the legal system may have difficulties engaging in treatment due to competing priorities, including meetings with lawyers and attending court hearings, as well as having more acute needs and treatment delays^34,35^, which can increase the risk of disengagement. Continued efforts to provide opportunities to patients with legal involvement to engage in EPI should be a priority, as the model has been shown to reduce criminal accusations^36^.

Consistent with past research^7^, medication nonadherence emerged as a predictor of early disengagement. This finding can be explained in a few ways: first, antipsychotic and mood stabilizing medication may cause intolerable side effects leading the patient to become nonadherent, and this negative experience sows mistrust, leading to service disengagement. This explanation is supported by survey findings, with patients and families reporting medication side effects as a top barrier to engagement. Second, patients who are nonadherent to medication decompensate and become too disorganized or lack the insight to engage in treatment^37^. Poor therapeutic alliance, medication side effects, and a lack of psychoeducation and insight into illness among patients have been identified as factors that can influence nonadherence in past studies^6,7,37,38^. Third, there may be common underlying factors, such as lack of insight or increased symptomology^7,39^, contributing to both nonadherence and disengagement. Nevertheless, these findings lend support for shared decision-making, leveraging standardized assessments of medication and side effects, psychoeducation on the risks of medication nonadherence, use of minimum effective dosing, and long-acting injectable formulations to improve medication nonadherence.

Use of the full range of recovery-oriented services in EPI was associated with lower rates of early disengagement, namely, IRT and SEE. IRT uses a strengths-based approach to psychotherapy, with a focus on shared decision-making, psychoeducation, illness self-management, and recovery goal setting^40^. Features of IRT and NAVIGATE more broadly, particularly the therapeutic alliance and perceived autonomy, have been associated with prolonged treatment participation^41,42^, but more research is needed to understand which aspects of IRT facilitate engagement. The impact of the therapeutic relationship on engagement was also salient in the surveys and likely contributes to the success of IRT. IRT provides an opportunity to build trust with patients and work through past negative experiences with services, which patient surveys identified as a barrier to engagement. Having a strong therapeutic relationship with patients in the context of IRT has been associated with improved mental health and quality of life outcomes^41^. However, this is contingent on patients participating in treatment and still raises questions about how to actively engage patients early in care.

The relationship between use of IRT and service engagement can also be explained by underlying patient factors, that is, the same patients who are likely to use IRT are likely to remain engaged in services. Nonetheless, few past studies have examined specific treatment components as influencing service engagement in EPI, whereas we found that if patients used these components early on, they were less likely to disengage. This suggests that standardizing care may buffer effects of health disparities so long as patients use the various components. It may also be helpful to implement a more targeted approach to identify, stabilize, and actively engage patients early in treatment who may be vulnerable to disengaging, using strategies to develop the therapeutic relationship, building motivation to use IRT through motivational interviewing, and providing education about and close monitoring of medication side effects.

Suggestions for improving engagement in EPI services were to offer appointments outside of traditional work hours, provide appointment reminders, more actively involve family members or other supports in treatment, and leverage digital tools, including text message and email communication. Interestingly, over half of patients and family members did not endorse virtual appointments as a suggestion to improve engagement (this survey was completed prior to the shift to virtual care amid the COVID-19 pandemic). These findings may be explained by concerns about confidentiality, losing in-person interactions with their care team, affordability, technology issues, or lacking digital literacy skills^43^. These perspectives may have now shifted as patients and families become more comfortable with virtual care as a feasible service delivery method^44^, especially for those who may not feel comfortable leaving the house, experience stigma coming to a hospital, or do not live close to services, all of which emerged as top barriers to engagement in the surveys. Virtual care has the potential to make appointments more flexible and maintain or improve appointment attendance^44–46^, but it is not a one-size-fits-all approach; a blended treatment model may benefit patients at different points in their care journey, though more high-quality trials are needed to examine the clinical effectiveness of virtual and blended models of care^47^.

Our study has several strengths, including the integration of survey and chart review data to better understand the complexities of disengagement. We also captured reasons for disengagement by distinguishing all-cause disengagement from early dropout from services and focused on the critical period early in treatment. However, we were not able to explicitly capture the experiences of patients who disengaged from services because they felt better and/or received what they wanted or needed from treatment, highlighting an area for future research. While it is unclear why some factors historically associated with disengagement did not have a substantive effect in our models, measurement issues may have contributed as we could only extract information recorded in patient charts. For example, problem substance use was captured through clinical documentation as standardized measures were not available. We also used a broad definition of family support, defined by presence of a family member at any appointment, which does not capture engagement quality.

Although our chart review relied mostly on structured assessments, extraction of information from narrative documentation was less reliable; for example, interrater reliability was considered moderate for medication nonadherence. Furthermore, diagnosis at consult was made by psychiatrists rather than structured diagnostic assessment, and therefore only reported as affective vs. nonaffective psychosis rather than specific diagnoses. This may be a missed opportunity given recent findings that a diagnosis of schizophreniform or brief psychotic disorder was associated with EPI service disengagement^48^. More structured ways of capturing key variables are needed; we chose to use chart review because of concerns that relying on participant recruitment and primary data collection would not capture those at highest risk of disengagement. Disengagement risk may also be better explained by other unmeasured factors, including standardized measures of symptom severity and functioning, which we were not able to include due to low completion rates. The Service Engagement Scale is a useful measure completed in the clinic that would have provided a continuous measure of engagement, but few were completed.

Our study suggests that some patients are vulnerable to disengagement early in EPI care. However, early engagement of patients in structured components of coordinated specialty care may help retain them, possibly buffering the risks of health disparities that contribute to disengagement. Focusing efforts on methods for managing medication side effects, encouraging use of IRT early in care to build the therapeutic relationship, and implementing digital strategies to help address practical challenges of attending appointments may help facilitate early engagement. Future studies may specifically target young people with psychosis who have disengaged from services to share their perspectives and contextualize the growing literature from observational data in this area.

## Supplementary information

Supplementary Table S1

Supplementary Table S2

Supplementary Table S3

Supplementary Table S4

Supplementary Table S5

## Data Availability

The data that support the findings of this study are available from the corresponding author, NK, upon reasonable request to protect the privacy and confidentiality of participants.
