# Supplementary material for: Disengagement from early psychosis intervention services: an observational study informed by a survey of patient and family perspectives
Source: Schizophrenia (Heidelb). 2022 Nov 11;8(1):94. doi: 10.1038/s41537-022-00300-5 (PMC9651118; doi:10.1038/s41537-022-00300-5)
Supplement: Supplementary file 2 — Supplementary Table S2 [file 41537_2022_300_MOESM2_ESM.docx]

**Supplementary Material**

**Supplementary Table S2.** Recoded variables

| Variables | Recoding |
| --- | --- |
| Gender | Male, female or other including intersex, trans, two-spirit or non-binary |
| Racial/ethnic group | Asian, Black, White, or other visible minority |
| Living situation | Living with or without family/romantic partners |
| Highest level of education | High school or less, attended some post-secondary, or completed post-secondary |
| Vocational activity | Full/part time work, full/part time school, or unemployed |
| NEET | Engaged or not engaged in employment, education, or training |
| Problem substance use | Problem substance use or no problem substance use |
| Diagnosis | Affective or non-affective psychosis |
| Referral source | Non-acute referral source or acute referral source including the ED or inpatient unit |

NEET, not engaged in employment education, or training; ED, emergency department.
