# Supplementary material for: Disengagement from early psychosis intervention services: an observational study informed by a survey of patient and family perspectives
Source: Schizophrenia (Heidelb). 2022 Nov 11;8(1):94. doi: 10.1038/s41537-022-00300-5 (PMC9651118; doi:10.1038/s41537-022-00300-5)
Supplement: Supplementary file 3 — Supplementary Table S3 [file 41537_2022_300_MOESM3_ESM.docx]

**Supplementary Material**

**Supplementary Table S3.** Patient- and family-reported top 5 facilitators and barriers to service engagement

| Variable | Top Patient (*n*=167) | Top Family (*n*=79) |
| --- | --- | --- |
|  | *n* (%) | |
| **Facilitators** |  |  |
| Location of services | 59 (35.3) | 26 (32.9) |
| Times services are offered | 49 (29.3) | 16 (20.3) |
| My clinician speaks with me about my personal goals and thoughts about treatment | 73 (43.7) | – |
| My clinician and I agree on what is important for me to work on | 57 (34.1) | – |
| I believe my clinician has an understanding of what my experiences have meant to me | 61 (36.5) | – |
| My clinician explained the rationale/reasons for services | 50 (29.9) | – |
| My clinician helped problem solve things that could get in the way of me attending | 16 (9.6) | 9 (11.4) |
| My clinician makes the effort to see me | 29 (17.4) | – |
| I am confident in my clinician’s ability to help me | 49 (29.3) | – |
| I believe that the treatments are helpful or will be helpful | 53 (31.7) | 24 (30.4) |
| I appreciate the collaborative approach to treatment | 36 (21.6) | 24 (30.4) |
| My family member helps get me there | 24 (14.4) | – |
| I don’t want to disappoint my family, friends or romantic partner | 52 (31.1) | – |
| My own motivation and commitment to treatment | 70 (41.9) | – |
| My past positive experiences with services | 23 (13.8) | 4 (5.1) |
| My family member has a positive impression of the clinician(s) | – | 34 (43.0) |
| I have a positive impression of the clinician(s) | – | 18 (22.8) |
| My family member, the clinician and I have established an understanding of the kinds of changes that would be good for my family member | – | 23 (29.1) |
| The clinician helped problem solve things that could get in the way of me attending appointments | – | 9 (11.4) |
| The clinician makes the effort to see my family member | – | 24 (30.4) |
| The clinician makes an effort to include me in my family member’s treatment | – | 16 (20.3) |
| My family member believes that the treatments are helpful or will be helpful | – | 29 (36.7) |
| I help my family member with reminders, transportation, etc. | – | 27 (34.2) |
| My family member doesn’t want to disappoint people | – | 19 (24.2) |
| My family member is motivated and committed to treatment | – | 29 (36.7) |
| My family member’s past positive experiences with services | – | 6 (7.6) |
| **Barriers** |  |  |
| Location of services | 31 (18.6) | 21 (26.6) |
| Times services are offered | 25 (15.0) | 15 (19.0) |
| The facilities are not inviting | 13 (7.8) | 6 (7.6) |
| I don’t like coming to a hospital | 40 (24.0) | 6 (7.6) |
| I am too busy | 29 (17.4) | 4 (5.1) |
| I forget appointments or lose track of time | 43 (25.7) | 4 (5.1) |
| I have too many appointments | 17 (10.2) | 3 (3.8) |
| Using drugs or alcohol | 8 (4.8) | 12 (15.2) |
| I do not like/trust my clinician | 14 (8.4) | 1 (1.3) |
| I believe my clinician withholds the truth from me | 13 (7.8) | – |
| My clinician is stern with me when I speak about things that are important to me and my situation | 11 (6.6) | – |
| My clinician is impatient with me | 9 (5.4) | – |
| There is a cultural/language barrier with my clinician | 6 (3.6) | 4 (5.1) |
| I do not agree with my clinician | 9 (5.4) | – |
| My clinician is too focused on medication | 28 (16.8) | 11 (13.9) |
| I get frustrated waiting for my clinician | 14 (8.4) | 2 (2.5) |
| It is uncomfortable when I disagree with my clinician or haven’t followed the treatment plan | 21 (12.6) | – |
| The services don’t feel relevant for my needs | 16 (9.6) | – |
| I don’t believe that the treatments are helpful or will be helpful | 22 (13.2) | 0 (0.0) |
| I am not ill/I do not require services | 9 (5.4) | – |
| I would prefer to address my problem without professional help | 12 (07.2) | 0 (0.0) |
| It is embarrassing to come for services | 23 (13.8) | 1 (1.3) |
| I have physical limitations that get in the way | 7 (4.2) | 2 (2.5) |
| My family, friends, or romantic partner doesn’t support me coming for services | 7 (4.2) | – |
| Past negative experiences with services | 35 (21.0) | 3 (3.8) |
| I am bothered by medication side effects | 48 (28.7) | – |
| I feel uncomfortable leaving my house or going to unfamiliar places | 31 (18.6) | 3 (3.8) |
| My family member does not like/trust the clinician | – | 14 (17.7) |
| The clinician does not make an effort to include me in my family member’s care | – | 6 (7.6) |
| My family member does not want me to be as actively involved in care as I would like to be | – | 8 (10.1) |
| I am not able to get information about my family member’s care due to consent laws | – | 8 (10.1) |
| My family member, the clinician and I do not agree on the best way to approach treatment | – | 4 (5.1) |
| My family member is bothered by medication side effects | – | 31 (39.2) |
| I get frustrated waiting for the clinician | – | 2 (2.5) |
| It is uncomfortable when my family member disagrees with the clinician or hasn’t followed the treatment plan | – | 8 (10.1) |
| The services don’t feel relevant for my family member’s needs | – | 3 (3.8) |
| My family member is not ill/does not require services | – | 0 (0.0) |
| My family member would prefer to address problems without professional help | – | 13 (16.5) |
| My family member feels uncomfortable leaving the house or going to unfamiliar places | – | 10 (12.7) |
