# Supplementary material for: Disengagement from early psychosis intervention services: an observational study informed by a survey of patient and family perspectives
Source: Schizophrenia (Heidelb). 2022 Nov 11;8(1):94. doi: 10.1038/s41537-022-00300-5 (PMC9651118; doi:10.1038/s41537-022-00300-5)
Supplement: Supplementary file 4 — Supplementary Table S4 [file 41537_2022_300_MOESM4_ESM.docx]

**Supplementary Material**

**Supplementary Table S4.** Patient- and family-reported suggestions to improve service engagement

| Suggestions for improvement | Patient (*n*=167) | Family (*n*=79) |
| --- | --- | --- |
|  | *n* (%) | |
| Appointments during evenings | 54 (32.3) | 30 (38.0) |
| Appointments on weekends | 57 (34.1) | 36 (45.6) |
| Appointments over the internet (like Skype) | 43 (25.8) | 26 (32.9) |
| More inviting facilities | 32 (19.2) | 19 (24.1) |
| Send appointment reminders | 61 (36.5) | 34 (43.0) |
| Communicate by phone | 35 (21.0) | 25 (31.7) |
| Communicate by text | 46 (27.5) | 29 (36.7) |
| Communicate by email | 49 (29.3) | 22 (27.9) |
| Clinician to offer appointments in the home | 23 (13.8) | 23 (29.1) |
| Clinician to offer appointments in the community (e.g., coffee shop, drop-in centre) | 32 (19.2) | 26 (32.9) |
| Clinician to change approach | 12 (7.2) | 8 (10.1) |
| More frequent appointments | 25 (15.0) | 13 (16.5) |
| Less frequent appointments | 17 (10.2) | 2 (2.5) |
| Longer appointments | 27 (16.2) | 10 (12.7) |
| Shorter appointments | 14 (8.4) | 0 (0.00) |
| Involve family members and other supports more | 22 (13.2) | 34 (43.0) |
| Involve family members and other supports less | 9 (5.4) | 0 (0.00) |
