# Supplementary material for: Disengagement from early psychosis intervention services: an observational study informed by a survey of patient and family perspectives
Source: Schizophrenia (Heidelb). 2022 Nov 11;8(1):94. doi: 10.1038/s41537-022-00300-5 (PMC9651118; doi:10.1038/s41537-022-00300-5)
Supplement: Supplementary file 5 — Supplementary Table S5 [file 41537_2022_300_MOESM5_ESM.docx]

**Supplementary Material**

**Supplementary Table S5.** Log-rank test comparing disengagement survival curves across groups for categorical variables

| Variable | Observed | Expected | *X*^2^ | *P* |
| --- | --- | --- | --- | --- |
| Gender (female) | 13 | 12.5 | 0.03 | .857 |
| Racial/ethnic group | – | – | – | – |
| Asian | 6 | 11.3 | 4.52 | .210 |
| Black | 8 | 8.58 | – | – |
| White | 13 | 10.6 | – | – |
| Other | 12 | 8.53 | – | – |
| Experienced homelessness | 12 | 8.66 | 1.65 | .200 |
| NEET | 21 | 22.8 | 0.35 | .556 |
| Living without family | 10 | 10.8 | 0.08 | .774 |
| Legal involvement | 13 | 7.09 | 6.01 | .014 |
| Family involvement in care | 26 | 27.5 | 0.27 | .601 |
| Problem substance use | 20 | 18.2 | 0.35 | .556 |
| Early medication nonadherence | 24 | 18.0 | 3.78 | .052 |
| Early use of IRT | 11 | 22.0 | 12.2 | .001 |
| Early use of SEE | 9 | 17.7 | 7.63 | .006 |

*X*^2^, chi-square statistic; NEET, not engaged in employment, education, or training; IRT, individual resiliency training; SEE, supported employment and education.
